# Machine Learning in Pediatric Healthcare: Current Trends, Challenges, and Future Directions

**DOI:** 10.3390/jcm14030807

**Published:** 2025-01-26

**Authors:** Hammad A. Ganatra

**Affiliations:** Pediatric Critical Care Medicine, Cleveland Clinic Children’s, 9500 Euclid Ave, Cleveland, OH 44195, USA; ganatrh@ccf.org

**Keywords:** artificial intelligence, machine learning, pediatrics

## Abstract

**Background/Objectives**: Artificial intelligence (AI) and machine learning (ML) are transforming healthcare by enabling predictive, diagnostic, and therapeutic advancements. Pediatric healthcare presents unique challenges, including limited data availability, developmental variability, and ethical considerations. This narrative review explores the current trends, applications, challenges, and future directions of ML in pediatric healthcare. **Methods**: A systematic search of the PubMed database was conducted using the query: (“artificial intelligence” OR “machine learning”) AND (“pediatric” OR “paediatric”). Studies were reviewed to identify key themes, methodologies, applications, and challenges. Gaps in the research and ethical considerations were also analyzed to propose future research directions. **Results**: ML has demonstrated promise in diagnostic support, prognostic modeling, and therapeutic planning for pediatric patients. Applications include the early detection of conditions like sepsis, improved diagnostic imaging, and personalized treatment strategies for chronic conditions such as epilepsy and Crohn’s disease. However, challenges such as data limitations, ethical concerns, and lack of model generalizability remain significant barriers. Emerging techniques, including federated learning and explainable AI (XAI), offer potential solutions. Despite these advancements, research gaps persist in data diversity, model interpretability, and ethical frameworks. **Conclusions**: ML offers transformative potential in pediatric healthcare by addressing diagnostic, prognostic, and therapeutic challenges. While advancements highlight its promise, overcoming barriers such as data limitations, ethical concerns, and model trustworthiness is essential for its broader adoption. Future efforts should focus on enhancing data diversity, developing standardized ethical guidelines, and improving model transparency to ensure equitable and effective implementation in pediatric care.

## 1. Introduction

The use of artificial intelligence (AI) and machine learning (ML) in healthcare has been rapidly evolving, offering transformative possibilities for improving patient care, diagnostics, and clinical decision-making. AI and ML are often used interchangeably, but they represent distinct concepts within the broader field of computational sciences. AI is a broad category that refers to the simulation of human intelligence in machines, enabling them to perform tasks typically requiring human cognition, such as reasoning, learning, problem-solving, and decision-making. ML is a subset of AI and focuses on developing algorithms that enable computers to learn from data and improve their performance on specific tasks without being explicitly programmed [1]. In the context of healthcare, AI encompasses a wide array of technologies, including expert systems, natural language processing, and robotics, while ML specifically leverages statistical and computational techniques to analyze large datasets, identify complex patterns, and make data-driven predictions or decisions. Leveraging ML models can also allow automated learning and adaptation, which is particularly powerful in dynamic environments like healthcare [2]. These technologies have demonstrated their potential to revolutionize the way healthcare is delivered by providing data-driven insights, enhancing predictive capabilities, and assisting healthcare professionals in delivering personalized treatment [3].

Pediatric healthcare presents unique challenges compared to adult care. Children are not simply smaller versions of adults; they have distinct developmental, physiological, and psychological needs [4]. Additionally, the medical data for children are often limited due to smaller sample sizes, ethical concerns, and variability across different developmental stages [4]. ML offers promising solutions to address these challenges by improving diagnostic accuracy, enhancing treatment strategies, and predicting outcomes tailored to pediatric patients.

In recent years, healthcare costs have increased significantly [5], while physician and nursing staff shortages have become more pronounced, particularly since the COVID-19 pandemic [6]. The increasing demand for healthcare services, coupled with limited human resources, has strained the system, affected the quality of care and increasing burnout among healthcare professionals [6]. AI and ML hold significant potential to improve efficiency, enhance patient outcomes, and alleviate the work burden faced by healthcare providers. By automating routine tasks, providing predictive insights, and supporting clinical decision-making, these technologies can help reduce the workload of healthcare professionals, decrease costs, and ultimately lead to better care delivery.

This narrative review is presented by a practicing pediatric intensivist with formal education in computer science and aims to provide an up-to-date overview of the current literature on ML in pediatric healthcare, highlighting applications, trends, opportunities, and challenges. Based on the existing literature, we explore the diagnostic, prognostic, and therapeutic roles of ML, discuss the limitations and ethical considerations, and outline future directions in which ML can make significant contributions to pediatric care.

## 2. Review Methodology

To conduct a narrative review on the topic of AI and ML as it pertains to pediatric healthcare, a comprehensive search of the PubMed database was performed. This review was limited to the PubMed database based on its strengths, including its extensive coverage of high-quality biomedical literature, robust search capabilities using Medical Subject Headings (MeSH), and its specific focus on healthcare-related research. The Boolean search query used was as follows: [(“artificial intelligence” OR “machine learning”) AND (“pediatric” OR “paediatric”)]. This search strategy was designed to capture all the relevant literature involving the use of artificial intelligence and ML in pediatric settings. The time frame was open-ended since ML is a relatively newer scientific field, and we wanted to see its evolution since introduction into pediatric care. The final search was performed on 23 November 2024.

For study selection, the inclusion criteria required the following: (1) original research articles focused on applications of machine learning in pediatric healthcare; (2) studies published in peer-reviewed journals; and (3) research that directly addressed diagnostic, prognostic, or therapeutic uses of ML. The exclusion criteria were as follows: (1) review articles, meta-analyses, systematic reviews, and commentaries; (2) studies not specific to pediatric populations or not involving machine learning applications; and (3) non-peer-reviewed publications or editorials.

**Figure 1:**
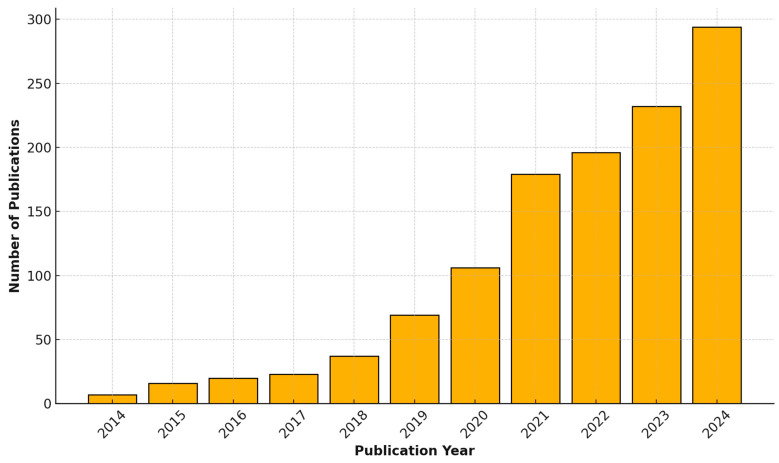
Publication trends for machine learning research in pediatric healthcare.

Our search strategy identified 1243 original peer-reviewed publications, with the earliest study on ML in pediatrics dating back to 1996. During the late 1990s and early 2000s, only a few preliminary studies were published, averaging one to three papers annually. However, starting in 2014, there was a significant increase in publication activity on this topic, with ten or more papers being published each year. The year 2024 is projected to set a record, potentially crossing 300 original studies that integrate pediatrics and ML. Consequently, this review focuses on publications during the decade from 2014 to 2024 (N = 1179). The publication trends over this period are illustrated in Figure 1.

The full texts of all the resulting papers were retrieved and reviewed by the author. Of the 1179 studies identified during this review process, only those that directly addressed the key themes, methodologies, and applications of ML in pediatric healthcare were included in the narrative synthesis. These publications were reviewed by the author, who applied domain knowledge to summarize significant advances in the field, offering a perspective on the current state of ML in pediatric healthcare. When multiple papers conveyed similar ideas and principles, only the most recent and representative studies were cited in this manuscript. Supplementary file S1 provides an exhaustive listing of all the identified studies to enhance transparency and offer a comprehensive resource.

In addition to identifying advances, this review also focused on uncovering gaps in the current research and discussing the challenges faced by ML applications in pediatric settings. By understanding these gaps and challenges, this review aims to provide insights into future research opportunities and the potential improvements that can be made to integrate ML effectively into pediatric healthcare.

## 3. Current Applications of Machine Learning in Pediatrics

ML has shown significant promise in a wide range of pediatric specialties, with applications including diagnostic support systems, prognostic modeling, therapeutic planning, and the management of specific pediatric conditions. Table 1 displays our summary review of the number of publications identified within particular pediatric subspecialty domains and the overarching themes of all the publications reviewed.

**Diagnostic support systems** have become increasingly integral in pediatric care, both in inpatient and outpatient settings. While most of these systems are currently based on rule-based AI with significant false alarms, the emerging literature indicates that ML can accurately diagnose complex pediatric conditions early in the disease process by analyzing clinical and demographic data. For instance, it has been demonstrated that ML plays a role in recommending appropriate genetic testing based on phenotype data, thereby enhancing diagnostic precision [7]. Convolutional neural networks have further enhanced diagnostic accuracy for pediatric brain tumors using MRI scans, outperforming traditional diagnostic methods. This improvement facilitates earlier interventions and better patient outcomes [8].

Similarly, studies have illustrated the use of ML in monitoring disease progression, predicting disease activity and relapse, and optimizing treatment strategies for pediatric Crohn’s disease patients [9]. In asthma management, ML has been used to analyze electronic health records (EHRs) and predict respiratory complications, enabling rapid interventions for high-risk children. This approach has reduced emergency department visits for asthma exacerbations, demonstrating its transformative impact on patient outcomes and healthcare efficiency [10,11]. Further expanding the scope of diagnostic support, researchers have constructed ML models that improve diagnostic accuracy for appendicitis, reducing the risk of missed or delayed diagnoses [12].

Another study employed an innovative technique to overcome the lack of pediatric sepsis data by normalizing pediatric physiological data to make them directly comparable with adult data and utilizing ML techniques for the early detection of pediatric sepsis, a leading cause of morbidity and mortality [13]. Notably, ML models have demonstrated the ability to predict sepsis onset earlier than clinical symptoms, enabling timely interventions and reducing mortality rates in pediatric ICUs [14]. Additionally, researchers have demonstrated machine learning’s ability to differentiate between benign and malignant causes of cervical lymphadenopathy, aiding in decision-making and reducing unnecessary invasive procedures [15], while others have emphasized its role in enhancing the reliability of diagnostic imaging by detecting anomalies that may be overlooked by human radiologists [16].

In various pediatric subspecialties, ML has been notably adopted in areas such as neurology, cardiology, and gastroenterology. For example, research has demonstrated machine learning’s capability to predict seizure status in neonates, facilitating early intervention and personalized care in a patient population in which EEG interpretation can be time-consuming and laborious [17]. In cardiology, ML models are employed to identify congenital heart defects and predict outcomes after cardiac events [18]. In gastroenterology, ML can be beneficial in managing chronic conditions like Crohn’s disease [9] and predicting the risk of bacterial gastroenteritis in children [19]. Additionally, studies have illustrated how ML models can assess nutritional deficiencies and recommend individualized dietary adjustments for children with chronic gastrointestinal conditions [20].

The application of ML extends to interpreting imaging data, analyzing electronic health records (EHRs), and incorporating wearable sensor data for outpatient care. ML in pediatric imaging, such as identifying abnormalities in chest X-rays or MRIs, is a growing focus area. In outpatient settings, wearable devices and mobile health technologies integrated with ML models provide continuous monitoring [21] and potentially early diagnosis of conditions like asthma and diabetes [22], bridging the gap between clinic visits and enabling proactive healthcare interventions [23].

**Advances in imaging modalities**, including narrow-band imaging, hyperspectral imaging, and multispectral imaging, have opened new frontiers in pediatric healthcare. These technologies, combined with ML algorithms, hold promise for enhancing diagnostic accuracy and enabling early disease detection. For instance, hyperspectral imaging can capture a wide range of wavelengths, providing rich datasets that ML models can analyze to identify subtle tissue changes indicative of pathology [24].

ML techniques have also improved the way CT scans are analyzed, making diagnoses more accurate while reducing radiation exposure for children. Advanced ML algorithms have been shown to segment and analyze pediatric CT images effectively, demonstrating good performance across diverse patient types and enabling the optimization of radiation doses [25]. Similarly, new approaches using ML are making MRI more accessible and affordable in settings with limited resources. ML has been shown to process images from low-cost MRI machines while maintaining high diagnostic quality, making this modality more feasible and affordable for widespread use in low-resource settings [26].

**Prognostic modeling** is a crucial application of ML in pediatric healthcare, as it aids in predicting patient outcomes, allowing for early intervention and better resource allocation. Several studies have highlighted the significant potential of ML models in predicting various clinical outcomes in pediatric patients. One of the most critical applications of prognostic modeling is predicting survival outcomes after severe clinical events. Research has developed predictive models to assess survival probabilities in pediatric patients who suffered cardiac arrest, leveraging features such as vital signs, lab results, and treatment data to provide real-time prognostic information that helps clinicians make informed decisions about patient management [18]. Predicting the length of stay (LOS) in pediatric settings is a valuable application for managing hospital resources efficiently, and attempts have been made to develop ML models that can accurately predict LOS in pediatric ICUs [27]. These predictions not only facilitate better planning and resource allocation but also contribute to improving patient management and ensuring smoother transitions in care.

**Personalized therapeutic planning** can also be accomplished by ML through the identification of optimal treatment strategies. Recent research demonstrates how ML algorithms recommend individualized medication dosing based on patient-specific factors, enhancing precision and reducing adverse effects [28]. Similarly, ML models can also be used to predict treatment responses. For example, studies have explored how ML could predict which pediatric patients with epilepsy would respond favorably to specific treatment protocols. This approach helps in individualizing treatment strategies, reducing the trial-and-error process typically involved in managing epilepsy and improving overall patient outcomes [29].

For young children with asthma, ML models have used clinical phenotypes to tailor interventions for children at high risk of asthma exacerbations, improving personalized care outcomes [30]. Similarly, wearable devices integrated with ML models for managing type 1 diabetes in children have shown improved glycemic control and reduced hypoglycemic events, resulting in individualized management and a significant enhancement in the daily life quality for children with diabetes [31]. ML models have also been applied in analyzing the variability in cost effectiveness of different therapeutic interventions, such as support modalities for children with respiratory failure, helping to optimize treatment plans for both improved patient outcomes and controlled healthcare costs [32]. Additionally, ML support has been demonstrated in managing critically ill pediatric patients undergoing continuous renal replacement therapy by predicting outcomes and adjusting treatment strategies based on patient-specific factors [33].

These applications underscore ML’s potential to transform pediatric care by enhancing diagnostic accuracy, predicting patient outcomes, and personalizing treatment plans. ML’s ability to learn from large datasets and make data-driven predictions is particularly valuable in pediatrics, in which individual variability and dynamic physiological changes pose significant challenges to traditional methods.

## 4. Trends and Common Techniques

**Popular Algorithms:** Machine learning in pediatric healthcare predominantly utilizes algorithms such as neural networks, random forests, support vector machines (SVMs), gradient boosting, and decision trees. Each algorithm offers specific advantages depending on the data type and healthcare problem addressed. For example, neural networks and deep learning models are commonly used for imaging data analysis [34], such as detecting abnormalities in MRI and X-ray scans, while decision trees and random forests are more frequently applied to structured data like electronic health records (EHRs).

**Figure 2:**
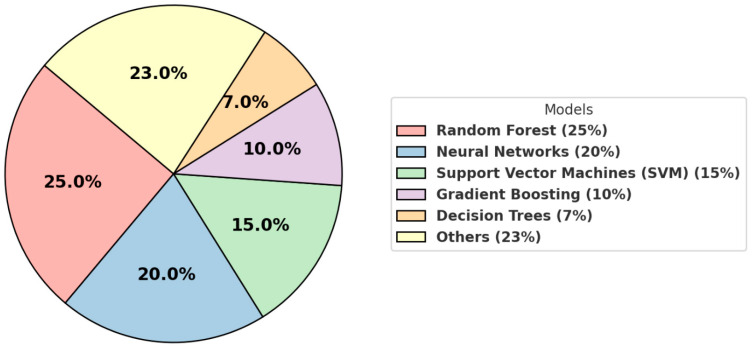
Major machine learning algorithms used and emphasized by reviewed publications.

Our review of the published literature for the last 10 years reveals that random forest models are the most commonly used, appearing in approximately 25% of the studies (Figure 2). Neural networks follow, representing around 20% of the applications, and support vector machines (SVMs) are referenced in about 15% of the studies. Gradient boosting and decision tree models are mentioned in roughly 10% and 7% of the studies, respectively.

Ensemble methods like random forest and gradient boosting are widely employed because they combine the outputs of multiple base models to improve robustness and accuracy, making them effective for a range of applications in pediatric healthcare. Taking a medical analogy, these methods act like a team of specialists collaborating to make a robust decision. For instance, random forest uses a collection of decision trees, each trained on different parts of the data, and averages their predictions to ensure reliable results. Gradient boosting, on the other hand, sequentially builds models, each focusing on correcting the errors of its predecessor, making it highly accurate but requiring careful tuning (Table 2).

Non-ensemble methods, including neural networks and SVMs, also contribute significantly to the landscape. Neural networks, inspired by the human brain, consist of numerous interconnected layers of mathematical computation that feed-forward and feed-backward to “learn” complex patterns from unstructured training data such as imaging (e.g., X-rays or MRIs). This is akin to a highly specialized expert identifying intricate abnormalities. SVMs, by contrast, are particularly effective for smaller datasets or those with many features, such as genetic markers or lab results, acting as precise classifiers that create boundaries between different classes of data.

**Data Types:** Machine learning models in pediatric healthcare utilize a wide variety of data types to make accurate predictions and improve clinical outcomes. Electronic health records (EHRs) are frequently used to capture longitudinal patient data, providing valuable insights for predicting health outcomes, and are leveraged in approximately 30% of the reviewed studies. Imaging data, including MRI and X-rays, are among the most widely used data types, employed in about 40% of the studies. These data types are crucial for diagnosing conditions like tumors, fractures, and congenital abnormalities. Studies utilizing convolutional neural networks have shown promising results in detecting tumors [35,36] and classifying chest X-rays [37,38,39] with high accuracy, aiding radiologists in early and precise diagnosis.

Physiological data, such as heart rate, oxygen saturation, and respiratory rate, are extensively used in predicting clinical deterioration in critically ill children, appearing in approximately 15% of the studies. Wearable sensors that collect continuous physiological data also play an important role in patient monitoring [40,41,42]. Genomic data, although less common, are increasingly used to understand genetic risk factors and personalize treatment strategies, involved in around 5% of the studies, particularly in oncology and rare genetic disorders. Wearable-device and sensor data from devices like activity trackers and glucose monitors feature in about 10% of the studies, primarily used for managing chronic conditions such as type 1 diabetes and asthma, allowing for personalized care and proactive intervention based on real-time data insights.

## 5. Challenges and Limitations

Despite the promising advancements, several challenges and limitations hinder the widespread adoption and effectiveness of ML in pediatric healthcare.

**Data limitations** are a primary concern, as ML models in pediatric healthcare are often hindered by issues related to data quality, availability, and small sample sizes. Pediatric datasets are typically smaller than those available for adult populations, limiting ML models’ ability to learn effectively and generalize [43]. It has been shown that accessing high-quality genetic data in pediatric care is challenging, with limited sample sizes impacting model reliability and accuracy [7]. Furthermore, variability in data collection methods and the lack of standardized datasets complicate data integration and model development. Studies have identified data heterogeneity as a significant obstacle for creating generalized ML models, as inconsistent data across institutions affects their accuracy and applicability in real-world clinical settings [32]. Additionally, limited data availability in specialized pediatric fields makes developing reliable models difficult, with small sample sizes and missing data being significant barriers [44]. Data sharing issues are also prominent, as privacy concerns restrict data sharing across institutions, leading to limited datasets for model training [45]. Although federated learning is suggested as a solution, its effectiveness depends on the quality and uniformity of data available at each site [45,46,47]. Moreover, the lack of standardization in data collection and reporting contributes to challenges in training ML models that generalize well across different settings, ultimately affecting their performance and reliability [48]. These challenges underscore the need for improved data-sharing practices, standardized data collection protocols, and initiatives to increase dataset sizes to ensure that ML models can effectively serve diverse pediatric populations.

**Ethical concerns** are paramount in pediatric ML research. Issues such as informed consent, data privacy, and the use of sensitive health information are critical when dealing with minors. It has been discussed that managing patient privacy, especially when handling genetic data, presents significant challenges [49]. The potential misuse of sensitive health information and the ethical implications of predictive modeling in vulnerable populations like children necessitate stringent regulations and ethical oversight. Additionally, the ethical dilemma surrounding the collection and use of genetic information in minors highlights concerns related to data ownership, informed consent, and the long-term implications of predictive genetic testing in children [7]. This underscores the need for robust ethical frameworks and policies. Furthermore, privacy challenges in specialized fields (e.g., pediatric urology) require extra layers of privacy protection and ethical considerations during data collection and use [44]. These issues collectively emphasize the importance of establishing comprehensive ethical guidelines to ensure the responsible use of ML in pediatric healthcare.

**Model generalizability** is another significant limitation, as ML models often struggle to perform well across diverse pediatric populations. Pediatric patients vary significantly in terms of age, development, and underlying health conditions, making it challenging to develop models that are broadly applicable. It has been highlighted that applying models trained on one population to another is difficult due to differences in demographics, healthcare settings, and data availability [50]. This limitation underscores the need for diverse and representative datasets to improve model robustness. Additionally, challenges in generalizing ML models to rare pediatric conditions have been identified, where limited data makes it difficult to develop models applicable across different populations [7]. Increased collaboration and data sharing across institutions are called for to enhance model generalizability and ensure equitable healthcare outcomes. Furthermore, the specialized nature of certain pediatric conditions poses difficulties in generalizing models, as the lack of data standardization across institutions hampers ML models’ performance in different healthcare settings, thereby limiting their applicability and reliability [44]. These issues emphasize the importance of creating comprehensive and standardized datasets and fostering collaborative efforts to enhance the generalizability of ML models in pediatric healthcare.

**Bias in data and models** remains a significant concern, as the limited availability of diverse pediatric data often results in models that do not perform well for underrepresented groups. It has been pointed out that, when genetic data are not representative of the broader pediatric population, there is a risk of bias, potentially leading to disparities in care in which certain subgroups receive less accurate predictions or recommendations [51]. Additionally, demographic biases have been highlighted, particularly when ML models are trained predominantly on data from high-income countries. This means that models trained on data from specific geographic regions may not be applicable to populations in other regions, leading to inequities in healthcare quality and outcomes [52]. Furthermore, the underrepresentation of minority groups in training data has been shown to result in biased risk stratification models, which can impact clinical decision-making and result in suboptimal care for underrepresented populations [53]. These issues underscore the importance of ensuring diversity in pediatric datasets and addressing demographic biases to develop fair and effective ML models in healthcare.

**Interpretability and trust** are crucial for the adoption of ML models in clinical practice, especially in pediatric settings in which the stakes are high. Black-box models, such as deep learning, often lack transparency, making it difficult for clinicians to trust their outputs. It has been emphasized that model interpretability is essential, as clinicians need to understand the rationale behind predictions to make informed decisions about patient care [52]. A lack of interpretability can hinder the acceptance of ML tools by healthcare professionals, limiting their practical utility. Additionally, the challenge of gaining clinician trust when using complex ML models for genetic test recommendations has been discussed, suggesting the incorporation of explainable AI (XAI) approaches to improve transparency and facilitate better clinician engagement with ML-generated insights [7]. Furthermore, the need for interpretability in specialized fields, in which clinicians may be less familiar with ML methods, has been highlighted [46]. By improving transparency, ML models can gain wider acceptance among healthcare providers, particularly in specialized and high-risk domains.

**Regulatory challenges** also pose significant barriers to implementing ML models in real-world clinical settings. Currently, there are no clear guidelines for the validation and approval of ML-based medical tools for pediatric care. While the regulatory environment is evolving, the absence of standardized frameworks for evaluating the safety, efficacy, and ethical implications of ML models remains a significant hurdle to clinical adoption. Federated learning involves training ML models across multiple institutions by sharing only the model and not the data and has been proposed as a safety mechanism to prevent data privacy leakage from ML research in healthcare. However, implementing federated learning in healthcare involves complex regulatory challenges, particularly in ensuring compliance with privacy regulations such as HIPAA [45]. There is a call for updated regulatory frameworks that can address the nuances of these novel ML training approaches. Additionally, the need for clear guidelines for approving ML models in specialized pediatric fields has been emphasized, as the lack of regulatory clarity makes it difficult for developers to navigate the approval process, delaying the integration of potentially beneficial tools [45]. These issues underscore the necessity for comprehensive and adaptable regulatory policies to facilitate the safe and effective adoption of ML technologies in pediatric healthcare.

**Practical barriers** to the real-world implementation of ML in pediatric healthcare remain unaddressed. The integration of ML models into existing EHRs and clinical workflows poses significant technical and logistical challenges. Many healthcare systems lack the infrastructure needed to support real-time data processing and the seamless deployment of ML algorithms at the bedside [54,55]. Additionally, the cost and scalability of integrating ML into resource-limited settings present further challenges. Healthcare facilities in such settings often operate with constrained budgets and limited technical capabilities, which hinder the adoption of advanced ML technologies [56]. Addressing these barriers will require the development of scalable, cost-effective solutions and collaborative efforts to integrate ML tools into routine clinical care.

## 6. Opportunities and Future Directions

**Emerging techniques** in ML continue to evolve, enhancing its effectiveness in pediatric healthcare. Deep learning, particularly convolutional neural networks, has shown promise in analyzing imaging data for diagnosing pediatric conditions such as brain tumors and respiratory diseases [57]. Federated learning enables multiple institutions to collaboratively train models without sharing sensitive patient data, thereby addressing privacy concerns [45]. Additionally, natural language processing (NLP) is being utilized to analyze clinical notes and extract valuable insights for improving pediatric care. For instance, studies have demonstrated that NLP can identify early warning signs from unstructured clinical notes, allowing for timely interventions [58]. These advancements illustrate the potential of innovative ML techniques to significantly improve diagnostic accuracy and patient outcomes in pediatric healthcare.

**Integrating ML into clinical practice** presents significant opportunities to enhance efficiency and outcomes in pediatric healthcare. Practical implementations have shown that ML models can assist in diagnosing life-threatening conditions and facilitating rapid treatment within routine clinical settings [59]. The use of wearable devices for continuous monitoring highlights how ML can support personalized care and provide real-time feedback to patients and caregivers [41]. Furthermore, predictive modeling can aid in planning discharges and optimizing resource allocation in pediatric intensive care units, facilitating smoother transitions in care [27]. These advancements demonstrate the potential of integrating ML into clinical workflows to improve patient outcomes and operational efficiency in pediatric settings.

**Research gaps** persist despite the advancements in ML applications within pediatric healthcare. The lack of diverse datasets, ethical considerations in data usage, and the interpretability of ML models require continued attention. Building on the solutions discussed earlier, fostering global collaborations and advancing XAI techniques will be crucial to addressing these challenges. Future research should also focus on scalable frameworks for collaborative data sharing and regulatory policies that support equitable AI deployment in pediatric care.

**Future reviews** could benefit from expanding the search to include additional databases to ensure a broader and more inclusive coverage of the field. This review was limited to the PubMed database based on its strengths; however, multidisciplinary databases such as Scopus or Web of Science, and engineering databases such as IEEE Xplore may contain additional relevant articles, particularly in fields like engineering or computer science, which intersect with healthcare.

## 7. Glossary

1. Artificial intelligence (AI): A branch of computer science focused on creating systems capable of tasks that typically require human intelligence, such as decision-making and problem-solving.
2. Machine learning (ML): A subset of AI that uses algorithms to identify patterns in data and improve performance on specific tasks without explicit programming.
3. Convolutional neural network (CNN): A type of deep learning model particularly suited to analyzing visual data, such as medical images.
4. Decision tree: A simple, interpretable model that splits data into branches based on feature values for classification or regression tasks.
5. Random forest: An ensemble learning technique that uses multiple decision trees to make predictions, improving accuracy and reducing overfitting.
6. Gradient boosting: A machine learning method in which models are built sequentially, with each one correcting the errors of the previous, often used for predictive tasks.
7. Support vector machine (SVM): A supervised learning algorithm that classifies data by finding the best boundary (or hyperplane) between classes.
8. Explainable AI (XAI): A set of tools and techniques to make the predictions and workings of machine learning models interpretable to clinicians and stakeholders.
9. Electronic health records (EHRs): Digital records of patients’ medical histories, treatment plans, test results, and other healthcare information.
10. Neural networks: A type of machine learning model inspired by the human brain, consisting of layers of interconnected nodes (neurons) that process data.
11. Black-box models: Machine learning models, such as deep learning, whose internal processes are not transparent or easily interpretable.
12. Bias (in machine learning): Systematic errors in models caused by non-representative or imbalanced datasets, potentially leading to unfair outcomes.
13. Federated learning: A technique allowing multiple institutions to train machine learning models collaboratively without sharing sensitive raw data.

## Figures and Tables

**Table 1:** Summary of pediatric subspecialties and machine learning research themes covered by original publications from 2014 to 2024.

	Count (Percentage)
**Pediatric Subspecialty**	
Radiology	225 (19.1%)
Genetics	139 (11.8%)
Infectious diseases	105 (8.9%)
Hematology/oncology	104 (8.8%)
Cardiology	99 (8.4%)
Surgery	90 (7.6%)
Neurology	85 (7.2%)
Gastroenterology/nutrition	84 (7.1%)
Respiratory/pulmonology	67 (5.7%)
Critical care	34 (2.9%)
Nephrology	23 (2.0%)
Administrative	22 (1.9%)
Endocrinology	19 (1.6%)
Psychiatry/mental health	16 (1.4%)
Neonatology	16 (1.4%)
Ophthalmology	14 (1.2%)
Orthopedic/musculoskeletal	12 (1.0%)
Multiple specialties	10 (0.8%)
Pharmacology	4 (0.3%)
Dentistry	4 (0.3%)
Obstetric/gynecology	3 (0.3%)
Emergency medicine	2 (0.2%)
Urology	2 (0.2%)
**Theme**	
Prognostics	833 (70.7%)
Diagnostics	767 (65.1%)
Screening	511 (43.3%)
Treatment	443 (37.6%)
ML methods	46 (3.9%)

**Table 2:** Applications and characteristics of machine learning algorithms in pediatric healthcare.

Algorithm	Primary Applications	Advantages	Challenges
**Random forest**	Predictive modeling, risk stratification	Robust, handles missing data well, interpretable.	May struggle with very high-dimensional data.
**Neural networks**	Imaging (e.g., X-rays, MRIs), diagnostics	Excels in complex data analysis, capable of identifying intricate patterns in imaging data.	Requires large datasets, computationally intense.
**Support vector machines**	Classification tasks	Effective with smaller datasets, performs well on high-dimensional data.	Limited scalability to large datasets.
**Gradient boosting**	Risk prediction, regression analysis	High accuracy, can handle mixed data types.	Prone to overfitting if not properly tuned.
**Decision trees**	Simple decision-making models	Easy to interpret, trains quickly on small datasets.	Prone to overfitting, low predictive power.

## Supplementary Materials

The following supporting information can be downloaded at: https://www.mdpi.com/article/10.3390/jcm14030807/s1, Database of original studies identified and reviewed.
